# Maternal Antibodies: Clinical Significance, Mechanism of Interference with Immune Responses, and Possible Vaccination Strategies

**DOI:** 10.3389/fimmu.2014.00446

**Published:** 2014-09-16

**Authors:** Stefan Niewiesk

**Affiliations:** ^1^Department of Veterinary Biosciences, The Ohio State University, Columbus, OH, USA

**Keywords:** maternal antibody, cotton rat, FcγRIIB, B cell receptor, maternal immunization

## Abstract

Neonates have an immature immune system, which cannot adequately protect against infectious diseases. Early in life, immune protection is accomplished by maternal antibodies transferred from mother to offspring. However, decaying maternal antibodies inhibit vaccination as is exemplified by the inhibition of seroconversion after measles vaccination. This phenomenon has been described in both human and veterinary medicine and is independent of the type of vaccine being used. This review will discuss the use of animal models for vaccine research. I will review clinical solutions for inhibition of vaccination by maternal antibodies, and the testing and development of potentially effective vaccines. These are based on new mechanistic insight about the inhibitory mechanism of maternal antibodies. Maternal antibodies inhibit the generation of antibodies whereas the T cell response is usually unaffected. B cell inhibition is mediated through a cross-link between B cell receptor (BCR) with the Fcγ-receptor IIB by a vaccine–antibody complex. In animal experiments, this inhibition can be partially overcome by injection of a vaccine-specific monoclonal IgM antibody. IgM stimulates the B cell directly through cross-linking the BCR via complement protein C3d and antigen to the complement receptor 2 (CR2) signaling complex. In addition, it was shown that interferon alpha binds to the CD21 chain of CR2 as well as the interferon receptor and that this dual receptor usage drives B cell responses in the presence of maternal antibodies. In lieu of immunizing the infant, the concept of maternal immunization as a strategy to protect neonates has been proposed. This approach would still not solve the question of how to immunize in the presence of maternal antibodies but would defer the time of infection to an age where infection might not have such a detrimental outcome as in neonates. I will review successful examples and potential challenges of implementing this concept.

## Introduction

Vaccination of neonates and infants is problematic because of two unsolved problems: the immature immune system of neonates and the presence of inhibitory maternal antibodies. A number of studies have determined that the immaturity of the immune system is most pronounced after birth and is overcome as the child develops. The immaturity (inability to fully respond to an antigenic stimulus) of the neonatal immune system has been observed in humans (1) and a number of other species, e.g., pig (2), cow (3, 4), and horse (5), and in experimental rodent models like mouse (1), rat (6), and cotton rat (7, 8).

Maternal antibodies are transferred from mother to child and protect neonates and infants during the time of maturation of their immune system. The vast majority of maternal antibodies are of the IgG isotype. In humans, maternal antibodies are preferentially transferred before birth transplacentally, and in animals of veterinary importance, preferentially through uptake of IgG in the intestine from colostrum within the first 24 h after birth. These passively acquired antibodies enter the bloodstream of offspring and act as a protective shield throughout the body in the same way as actively produced antibodies. Sometimes IgA antibodies contained in breast milk are also referred to as maternal antibodies. However, there are important differences in the action of passively transferred IgG and IgA antibodies. Upon transfer after birth, IgG antibodies are present in the bloodstream of the neonate in a finite amount that declines over time. These IgG antibodies suppress vaccine-induced immune responses. In contrast, IgA antibodies are continuously supplied through breast milk from the mother and protect the gastro-intestinal tract against pathogens without having an effect on the immune response. For the purpose of this review, the term “maternal antibodies” will be used for passively transferred IgG antibodies.

Maternal antibodies are very effective in protecting neonates and infants against most infectious diseases. The most impressive example is the protection of children with agammaglobulinemia (deficiency in the production of antibody) against bacterial infection for up to 6 months (9). Other documented examples of the ability of maternal antibodies to fully or partially protect are the amelioration of infection with respiratory syncytial virus (RSV) (10) or influenza virus (11) in humans, canine distemper virus in dogs (12), and infection with avian leukosis virus in chickens (13). Over time, maternal antibody titers decline because antibodies are being metabolized and do not protect any longer. However, even low, non-protective titers of maternal antibodies are still able to inhibit vaccination against infectious diseases of humans and animals. It is this phase of decaying maternal antibodies that presents a window of opportunity for infection by pathogens encountering the neonatal child or animal.

## Inhibition of Vaccination by Maternal Antibodies

In humans, maternal antibodies wane over a period of 6–12 months (14–17). The kinetics of maternal antibody decline is correlated to the amount of maternal antibody present in the neonate after birth in that higher titers persist for a longer time. In contrast to humans, the duration of maternal antibodies in agriculturally important animal species is usually 3–6 months (18–20) and in chicken only 4–7 days (21). In contrast, maternal antibodies in bats persist similar to humans for 6–12 months (22–24). Maternal antibodies in all species have been reported to reduce or abolish antibody generation after vaccination. The reduction or lack of antibody typically results in reduced or absence of protection against disease (Tables 1 and 2). It is of interest to note that all types of vaccines (live-attenuated, inactivated, subunit, and experimental vaccines) have been reported to be inhibited. For a number of experimental vaccines immunization with variable success in the presence of maternal antibodies has been reported but these reports have to be evaluated based on criteria set out in Section “Evaluation and Development of Experimental Vaccines.”

**Table 1:** **Inhibition of seroconversion of human vaccines by maternal antibodies**.

Infectious agent	Type of vaccine	Reference
Tetanus	Combination protein vaccine	(25)
Pneumococcus	Combination protein vaccine	(25, 26)
Hib	Combination protein vaccine	(25, 27)
Pertussis	Combination protein vaccine	(25)
	Acellular and whole-cell vaccine	(28)
Measles virus	Live-attenuated	(29–31)
Mumps virus	Live-attenuated	(32)
Hepatitis A virus	Inactivated virus	(33)
Hepatitis B virus	Protein vaccine	(34)
Rotavirus	Live-attenuated	(35)
Poliovirus	Inactivated virus	(36, 37)
	Live-attenuated vaccine	(38)
Influenza virus	Cold recombinant influenza and trivalent inactivated virus	(39)

*This table lists examples of studies, which document the inhibition of or reduction in seroconversion after immunization with both live and non-live vaccines. Jones et al. (25) document a stronger inhibitory effect of maternal antibodies on tetanus and pneumococcal vaccines than Hib and pertussis vaccines. Most studies indicate that higher levels of maternal antibodies inhibit antibody development more severely than low titers. Two studies (29, 35) indicate that an increase in dose helps to improve antibody responses after immunization in the presence of maternal antibodies*.

**Table 2:** **Inhibition of seroconversion of veterinary vaccines by. maternal antibodies**.

Species	Infectious disease	Type of vaccine	Reference
Dog	Canine parvovirus	Live-attenuated	(40, 41)
	Canine distemper virus	Live-attenuated	(42, 43)
Cat	Feline panleukopenia virus	Live-attenuated	(44, 45)
	Feline herpesvirus 1	Inactivated virus	(44)
	Feline calicivirus	Inactivated virus	(44)
Cow	Bovine viral diarrhea virus	Live-attenuated	(46, 47)
	Foot and mouth disease virus	Inactivated virus	(48)
	Bovine respiratory syncytial virus	Live-attenuated	(49–51)
Pig	*Erysipelothrix rhusiopathiae*	Live-attenuated	(52)
	Pseudorabies virus	Genetically attenuated	(53)
	Classical swine fever virus	Protein vaccine	(54, 55)
		Live-attenuated	
	Influenza virus	Protein vaccine	(56)
Chicken	Influenza virus	Inactivated virus	(57)
Raccoon	Rabies virus	Vaccinia virus expressing rabies glycoprotein	(58)
	Canine distemper virus	Live-attenuated	(59)
Wolves	Canine distemper virus	Live-attenuated	(60)
Ferrets	Canine distemper virus	Live-attenuated	(61)

*This table lists examples of studies, which document the inhibition of or reduction in seroconversion after immunization with both live and non-live vaccines in different species. It also lists an example of a vaccine (against canine distemper virus), which is inhibited in several species. Three studies (46, 51, 53), included in their study T cell measurements and detected T cell responses after immunization in the presence of maternal antibodies although the antibody response was inhibited*.

## Example: Inhibition of Measles Vaccination by Maternal Antibodies

The best studied example in human and veterinary medicine for the inhibition of vaccination by maternal antibody is measles vaccination. The measles vaccine virus (Edmonston strain) was developed in the late 1950s and early 1960s by attenuating a wild type virus on human and chicken embryo fibroblasts [for review see Ref. (62)]. Today various derivatives of the Edmonston strain are used as vaccine viruses worldwide (63). Immunization by both the standard subcutaneous and more experimental respiratory routes has been successful in protecting seronegative children (64). The protective immune response induced by vaccination consists of neutralizing antibodies against the two glycoproteins, with the (receptor-binding) hemagglutinin protein being the major target and the fusion protein the minor one (65). In addition, CD4 as well as CD8 T cell responses are induced. Case reports describing persistent MV infection in T cell deficient patients support the notion that T cells are required to clear infection but do not protect against infection (66). This role of CD8 T cells in virus clearance has been confirmed in the Rhesus macaques model (67). In cotton rats, CD4 T cells have no role in protecting or clearing virus from the respiratory tract (68). However, in mice they have a role in clearing virus infection from brain tissue through production of IFNγ (69, 70).

During their first year of life, children are protected by neutralizing maternal antibodies against MV infection. Over time, these antibody titers wane and eventually do not protect against wild type infection [for review see Ref. (71)]. Even these low, non-protective antibody titers inhibit seroconversion after both subcutaneous and intranasal immunization in cotton rats as well as humans. The lack of seroconversion severely reduces protection after vaccination with the live-attenuated vaccine virus. In the cotton rat model, this leads to a complete lack of protection (68). In clinical studies, immunization in the presence of maternal antibodies leads to partial reduction in mortality (72) and morbidity (73) (even in seronegative children). However, a broad clinically desirable protective immunity (protective T and B cell responses, and no clinical symptoms after infection rather then reduction in morbidity and mortality) is not established after immunization in the presence of maternal antibodies.

In contrast to the antibody response, MV-specific T cell proliferation is usually measurable after immunization in the presence of maternal antibodies (74–76). In spite of the inhibition of seroconversion, it was observed that vaccination in the presence of maternal antibodies leads to priming of B cells. Although no antibodies were produced, children immunized with measles vaccine in the presence of maternal antibodies responded with a higher immune response to booster vaccination after maternal antibodies had declined than children who had not been vaccinated at all (77). This study indicated that inhibition of B cell responses by maternal antibodies is a temporary effect and not due to the induction of anergy. However, it has also been observed that in children who were immunized in the presence of maternal antibodies long term antibody titers were reduced (even after boosting) compared to children who were seronegative at the time of immunization (76).

Since no current vaccine formulation is fully effective in the presence of maternal antibodies, two approaches have been used clinically to address the problem: one, the use of a high titer vaccine, and the other, the determination of the earliest time point possible for successful vaccination. The high titer vaccine (>10^4.7^ pfu) had a 10- to 50-fold higher viral titer than the normal vaccine and induced some level of protection after immunization in the presence of maternal antibodies (29, 78). However, the use of this vaccine was associated with increased mortality (particularly in females) (79–81), which was attributed to immune suppression by the vaccine and its use was discontinued. A possible explanation for higher mortality in girls might be that girls have been reported to have lower maternal antibody titers (82) that were overwhelmed by a higher vaccine virus dose.

In a second approach, children were immunized at different times after birth (in the face of declining maternal antibodies). These studies have shown that a low level of maternal antibody correlates best with vaccination success. At the age of 6 months, maternal antibody titers are still high enough to suppress seroconversion but at the age of 9 months vaccination campaigns are relatively successful (31, 83). However, the complete disappearance of antibody at the age of 12 months seems to be optimal for immunization (32, 74, 84–86). Additional studies into the levels of maternal antibodies demonstrated that strong regional differences exist in that in a number of countries maternal antibodies might have disappeared by 6 months of age, and sometimes immunization at 4.5 months of age might be partially successful (87, 88). Regional differences might be due to a number of factors such as the exposure to wild type virus infection of mothers, which results in higher antibody titers and higher transfer rates into children than in vaccinated mothers (89). Maternal antibody levels in children of vaccinated mothers are lower and decline earlier than in children from naturally infected mothers.

## Clinical Relevance of Inhibition of Vaccination by Maternal Antibodies and Practical Solutions

Numerous reports detail the inhibition of antibody responses after vaccination in the presence of maternal antibodies. The important question is whether this is clinically relevant and whether clinical solutions exist to overcome the problem. For measles virus vaccination, maternal antibodies clearly inhibit the generation of neutralizing antibodies and therefore severely restrict protective immunity. However, the clinical outcome might vary depending on the specific vaccine and infectious disease under study. A recent study found that suppression of antibody responses against the tetanus and pneumococcal vaccines was more pronounced than against hemophilus influenza B and pertussis vaccines (25). Theoretically, immunization should be successful when maternal antibodies have declined below the threshold of detection. In practice, it is not feasible to accurately predict this time point as it depends on the amount of maternal antibody transferred, region, gender, nutritional status, and species. Therefore, the measurement of antibody levels would be required before immunization, which is not feasible in a clinical setting. The clinical practice to immunize children and animals repeatedly because most immunizations are based on a prime-boost principle, also ensures that children and animals are immunized when maternal antibodies have been metabolized. Immunization in the presence of maternal antibodies does not interfere with later vaccinations, and this practice ensures that eventually the individual is immunized when maternal antibodies have disappeared. However, a variable period of time without active immune protection will remain and therefore this vaccination schedule is most successful in regions with a low prevalence of infection. The other important question for vaccination is whether antibodies or T cells are a correlate of protection. If T cells play a major role in protection [e.g., Aujeszky virus (90)], early immunization will afford protection because the T cell response is affected very little by maternal antibodies. If, in contrast, neutralizing antibodies are required for protection, early immunization is not likely to succeed.

## Type of Placenta Determines Mode of Transfer of Maternal Antibodies

The transfer of maternal antibodies from mother to offspring may occur during pregnancy (from the maternal blood via transplacental transfer) and within 24 h after birth (from colostrum via the small intestine). The amount of antibody transferred by these two mechanisms differs by species depending on the type of placenta (http://placentation.ucsd.edu/placenta.html). Just prior to formation of the placenta, there are a total of six layers of tissue separating maternal and fetal blood. There are three layers of fetal extraembryonic membranes in the chorioallantoic placenta of all mammals, all of which are components of the mature placenta: endothelium lining allantoic capillaries, connective tissue in the form of chorioallantoic mesoderm, and the chorionic epithelium, the outermost layer of fetal membranes derived from trophoblasts. There are also three layers on the maternal side, but the number of these layers that are retained – that is, not destroyed in the process of placentation – varies greatly among species (see Table 3). Overall, species with few layers between maternal and fetal blood have a higher rate of transport of maternal antibodies transplacentally [through Fc-receptors (FcR)]. In species with more layers between maternal and fetal blood, the transport of maternal antibodies preferentially occurs through colostrum. Although the differences between species are important for the study of placentation and transfer of nutrients, for the study of vaccination in the presence of maternal antibodies the structure of the placenta and the mode of transmission of IgG is only relevant if intrapartum studies are performed. Most studies, however, assess the inhibition of vaccination sometime after birth and the relevant parameter in these experimental settings is the amount of IgG being transferred to the offspring.

**Table 3:** **Type of placenta is species specific and determines route of transfer of maternal antibodies**.

Species	Placenta	Maternal antibody transfer	Transfer mediated by neonatal Fc receptor (FcRn)	Reference
Human	Hemochorial	Transplacental	Yes; preferential transport of IgG1 > IgG3 > IgG4 > IgG2	(91–94)
Rodents	Hemochorial	Transplacental/colostrum	Yes	(6, 95–98).
Mouse				
Rat				
Cotton rat	Hemochorial	Transplacental/colostrum	Unknown	(99, 100)
Dogs and cats	Endotheliochorial	Low transplacental/high in colostrum	Unknown	(12, 20, 40, 101–104)
Cattle, sheep, pigs, and horses	Epitheliochorial	Colostrum	FcRn present in pig intestine, role in IgG transfer questionable	(94, 105–107)
Birds	None	In ovo	FcRY (bird equivalent to FcRn)	(108, 109)

*The three potential maternal layers in a placenta are the endothelium lining of endometrial blood vessels, connective tissue of the endometrium, and endometrial epithelial cells. In humans and rodents, the fetal chorionic epithelium is in direct contact with maternal blood because only the maternal endothelium remains (hemochorial placenta). In contrast, the chorionic epithelium of horse and pig fetuses remains separated from maternal blood by three layers of tissue (epitheliochorial placenta) whereas only two layers remain in dogs and cats (endotheliochorial placenta). The fewer the layers between maternal and fetal blood the higher the rate of transport of maternal antibodies transplacentally. In humans, the majority of maternal IgG is transferred via the placenta. The transfer is an active process during which IgG binds to the neonatal Fc receptor (FcRn), which is located in the placental syncytiotrophoblast. The binding between FcRn and IgG is 100-fold higher at pH 6 than at pH 7.4 (91, 92). After endocytosis of IgG by the placental syncytiotrophoblast IgG binds to FcRn in the endosome at low pH, is transported to the opposite cell membrane and released at physiological pH into the blood stream [reviewed by Palmeira et al. (110)]. This mechanism explains why maternal antibodies are of the IgG isotype. In contrast to this specific transport mechanism, it seems that in agricultural species IgG and other macromolecules are transported from colostrum across the intestinal barrier in a non-specific fashion (111, 112). Within 24-h after birth, the intestine becomes impermeable to macromolecules and IgG transfer ceases. Similar to rodents, e.g., pig intestinal epithelial cells also express FcRn during the neonatal period but it has been argued that the large amount of IgG taken up within 24 h is not consistent with receptor-mediated transport [discussed in Ref. (107)]. In contrast to rodents, pig FcRN also is expressed in the adult period of life (106) and this expression pattern is not correlated with transfer of maternal antibody. In contrast to mammals, birds generate IgY antibodies instead of IgG antibodies and use the IgY Fc-receptor (FcRY) to transport IgY into the egg*.

## Experimental Models to Study the Effect of Homologous Versus Heterologous Passively Transferred Antibodies on Vaccination

Inhibition of vaccination has been studied in the presence of natural maternal antibodies or in the presence of passively transferred homologous or heterologous antibodies. Overall, inhibition of seroconversion after vaccination has been similar using both methods. For the induction of natural maternal antibodies, dams are immunized during pairing with the male and will transfer maternal antibodies to the pups. In rodents, differences can be found in antibody levels between pups of the same litter, which may occur because of the suckling hierarchy. The supposed advantage of natural maternal antibodies is the fact that antibodies from the same species have a predetermined fit for the FcR present in the species whereas heterologous IgG does not always interact with FcR (113, 114). However, studies in mice and humans have shown that subclasses of homologous IgG differ in their interaction with FcR (115, 116).

Often, the question of inhibition of vaccination by maternal antibodies will have to be separated from the immaturity of the neonatal immune system experimentally. One possible resolution is the transfer of homologous immune serum into adolescent animals (117). Using this experimental model, it was shown that suppression of vaccine responses correlates with amount of antibody present at the time of immunization (118). The injection of antibodies allows us to determine the amount of antibody transferred and ensures equal titers in all animals of the experimental group. In addition, often higher titers of passively transferred antibody can be achieved than would be possible through the induction of natural maternal antibodies. A disadvantage of homologous antibodies (whether natural maternal antibodies or passively transferred) is that they are indistinguishable from antibodies induced actively via immunization. The transfer of heterologous antibodies provides the advantage that actively induced and passively transferred antibodies can be distinguished by ELISA. Heterologous antibodies degrade faster than homologous antibodies, and e.g., in the cotton rat model the half-life of cotton rat IgG was estimated to be 7 days versus a half-life of 3.5 days for injected human IgG (Niewiesk, unpublished). The rate of decay should not matter for vaccination studies, as the amount of antibody at the time of vaccination is important for inhibition, not the duration of antibody levels. However, one should not inject heterologous antibodies repeatedly, as that will lead to the induction of regulatory T cells, which might interfere with the immune response after vaccination (119). Another caveat is warranted when monoclonal antibodies are used. It is important to test whether specific subclasses will interact with the FcγRIIB receptor in the respective species and be able to suppress B cell responses. In cotton rats, for example, mouse IgG1 antibodies do not bind to the FcγIIB receptor and do not interfere with vaccination whereas mouse IgG2a antibodies do (114).

## Mechanisms of Inhibition of Vaccination by Maternal Antibodies

### Antibody feedback mechanism

Since studies to address the mechanism of inhibition of seroconversion after vaccination in the presence of maternal antibodies have been rare, data from antibody feedback regulation have been used to provide mechanistic insight into B cell inhibition by antibody. Antibody feedback regulation is the phenomenon whereby co-injection of an antigen [usually sheep red blood cells (SRBC)] and a monoclonal antibody specific for this particular antigen into a naive mouse leads to a reduction or inhibition in the generation of antigen-specific antibodies. [It is important to note that this phenomenon is true for large particulate antigens like SRBC and keyhole limpet hemocyanin (KLH)]. The suppression increases with the amount and affinity of antibody given (120, 121) but is independent of the IgG isotype (120, 121). In contrast to the B cell response, a T helper cell response is not inhibited (122). Moreover, a recall response can be induced after the decline of passively transferred antibody. Two major hypotheses might explain this phenomenon: epitope masking and B cell regulation through the Fcγ-receptor IIB (FcγRIIB).

#### Inhibition of B cell responses through epitope masking

Sheep red blood cell-specific antibodies recognize very few highly repetitive epitopes on SRBC. The theory of epitope masking postulates that antibodies can cover these epitopes and mask them from B cell recognition if enough antibody is used. Originally, it was thought that all epitopes have to be covered by antibodies to prohibit recognition by B cells (epitope-specific suppression). However, it was shown that a monoclonal antibody against one epitope can suppress recognition of a whole particulate antigen by B cells (epitope unspecific suppression) (120, 121, 123). It has been argued that at high antibody concentrations, steric hindrance might explain this form of suppression. But even low antibody concentrations of 0.4 μg (corresponding to 2 × 10^6^ antibody molecules) are able to suppress antibody responses against 10^8^ SRBC (124). Epitope masking as a mechanism of suppression has always been discussed in conjunction with a competing mechanism, the regulation of B cells through the FcγRIIB.

#### Inhibition of B cell responses by binding of IgG to the Fcγ-receptor IIB

B cells express the FcγRIIB as the only Fcγ receptor on their surface. It has been shown *in vitro* that IgG specific for the B cell receptor (BCR) binds to the BCR via its antigen binding domain in the variable region and to FcγRIIB through the constant region. Through the cross-linkage between BCR and FcγRIIB, the tyrosine-based inhibitory motif of FcγRIIB is in close proximity to the tyrosine-based activation motif of the BCR. This proximity leads to inhibition of antigen-specific B cell activation. *In vitro*, SRBC-specific B cells from spleens of immunized mice can be activated by addition of SRBC to secrete antibody, which can be detected using an ELISPOT system. This activation can be inhibited by the addition of SRBC-specific IgG (124), which forms a complex with SBRC and links the BCR to FcγRIIB. In contrast, the F(ab′)_2_ fragment, which lacks the constant region (Fc) of IgG, cannot cross-link these receptors and inhibit B cell stimulation. Similarly, mouse B cells with a deletion in the gene coding for the γ chain of the FcγRIIB cannot be inhibited by SRBC-specific IgG (124). These *in vitro* data strongly argue for a role of FcγRIIB in down-regulating B cell responses. In contrast, data obtained *in vivo* do not unequivocally support the ability of IgG to suppress B cell responses by FcγRIIB binding. In support of this mechanism are data demonstrating that glycosylation of the constant region (Fc), which is crucial for binding of IgG to the inhibitory FcγRIIB, is necessary for inhibition (125, 126). In addition, some studies have shown that F(ab′)_2_ fragments, in contrast to complete IgG, do not inhibit antigen-specific responses (120, 127, 128). However, other studies found little difference between F(ab′)_2_ fragments and complete IgG (120, 127, 128), and in mice with a genetically deleted FcγRIIB (and also deletion of FcγRI and FcγRIII) inhibition still could be induced by IgG (124). Two technical comments must be made in regard to the latter studies. After pepsin digestion, the quality of the F(ab′)_2_ fragments varies and was not rigorously controlled in these studies. In the study using genetically modified mice, the deletion of the common γ-chain (FcRγ) leads to the absence of FcγRI, FcγRIIB, and FcγRIII. In consequence, these mice displayed a wide array of immunological abnormalities that were not restricted to the B cell compartment (124). After antigen injection, these mice generated a markedly stronger antibody response indicating the lack of a feedback mechanism to regulate B cell responses and antibody titers.

#### Stimulation of B cell responses by the binding of IgM to the CD21/CD19/CD81/Leu-13 signaling complex

In the study of antibody feedback regulation, it was found that co-injection of antigen and antigen-specific IgM increases antibody responses in the presence of an inhibitory IgG (125). These data suggest that the inhibitory signal through FcγRIIB can be overcome by stimulating signals via the complement receptor 2 (CR2) (CD21/CD19/CD81/Leu-13) complex. *In vitro*, a complex of antigen, IgM and complement protein C3d cross-links the BCR and CR2 and leads to B cell activation (129, 130). C3d which binds to CD21 is produced through the activation of the classical complement pathway involving the C2 and C4 complement proteins. *In mice*, the cross-linkage increases the responsiveness of B cells in the presence of antigen-specific IgM and antigen by two orders of magnitude in comparison to antigen alone (131). This immune enhancement by IgM is not seen in CD21 gene-targeted mice (132) or mice in which complement was depleted prior to vaccination (125) or in the absence of antigen. These data support a regulatory role of IgG on B cell activation that can be overcome by stimulation through IgM, and argues against simple epitope masking.

### Mechanism of inhibition of seroconversion after vaccination in the presence of maternal antibodies

In addition to mechanisms based on the work performed in the antibody feedback mechanism model (epitope masking and B cell inhibition through FcγRIIB), two additional mechanisms have been hypothesized to play a role in inhibition of vaccination by maternal antibodies: removal of vaccine antigen by macrophages and neutralization of vaccine virus by maternal antibodies.

#### Antigen removal by macrophages

It has been assumed that macrophages remove antibody–virus complexes from the circulation through binding to FcR to such a degree as to abolish immune responses. There is no experimental evidence to support this hypothesis, and it also does not explain why the B cell response is preferentially inhibited whereas a T cell response is consistently detected after immunization in the presence of maternal antibodies. The only evidence that Fc-receptor-mediated phagocytosis of antigen–antibody complexes redirects the immune response is the finding in mice that it leads to increased IL10 secretion (133). The increase in IL10 could direct T cell responses toward a Th2 response, which, however, would not lead to a decrease in antibody responses. So far, there has been no evidence that the removal of antigen–antibody complexes by macrophages influences antibody responses.

#### Neutralization of live-attenuated vaccine

An often-suggested explanation for the lack of vaccination success in the presence of maternal antibodies is neutralization of the vaccine virus, which would reduce the amount of viral antigen below a certain (undefined) threshold and thereby interfere with immune recognition. Three facts argue against this hypothesis: maternal antibodies suppress not only replicating live-attenuated vaccines but also (non-replicating) protein vaccines; immunization with vector systems expressing measles virus proteins (which are not sensitive to neutralizing antibodies) are inhibited by maternal antibodies (134–136); and non-neutralizing antibodies block vaccination with a live-attenuated vaccine (114).

#### B cell inhibition through epitope masking

The idea of *epitope masking* predicts that B cell epitopes on a vaccine will be covered by antibody and therefore will not be recognized by B cells. In consequence, this effect is dependent on the concentration of antibody present in the circulation, and should be seen with both a complete IgG antibody and an IgG antibody lacking its constant region [so-called F(ab)_2_ fragment]. However, experimentally we could demonstrate that one antibody at a high concentration is less efficient in inhibiting vaccination than three antibodies at lower concentrations, and that only complete IgG antibody can block vaccination (114). In addition, the inhibition of antibody generation afterward was not specific to the epitope recognized by the inhibitory antibody.

#### B cell inhibition through cross-link of BCR with FcγRIIB

**Figure 1:**
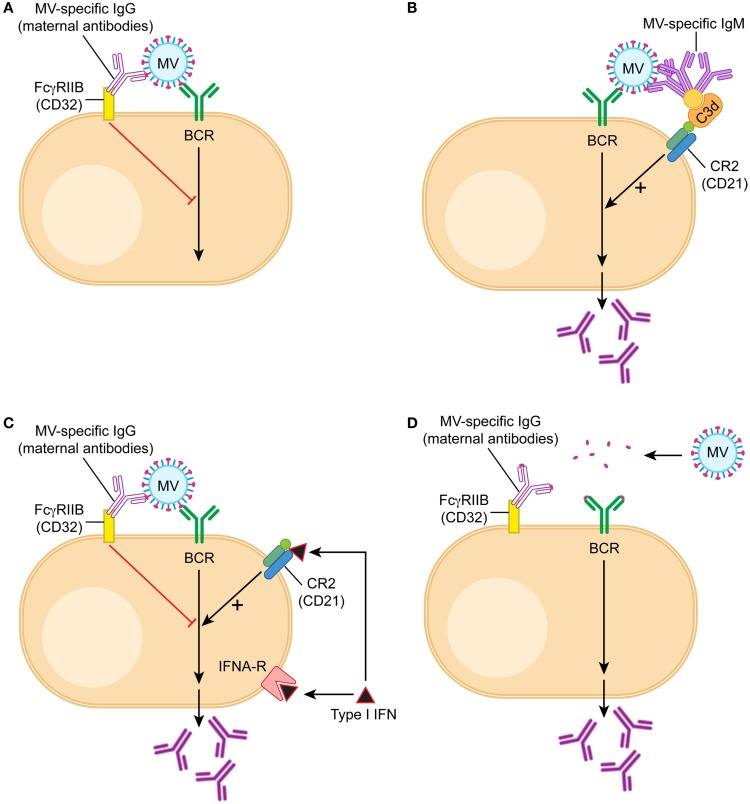
**Model of B cell activation in the presence of maternal IgG**. B cells are being stimulated through three signals, the first one is recognition of antigen by the B cell receptor (BCR), the second the interaction with T cells through CD40/CD40 ligand, and the third cytokines like type I interferon or IL-6. During vaccination in the presence of maternal antibodies T cell responses are generated and therefore the second signal is provided. **(A)** In the presence of maternal antibodies (IgG), the first signal is downregulated by a cross-link between BCR and FcγRIIB. If MV-specific IgG binds to MV, the constant region is bound by the receptor for the constant region (Fc) of IgG (which is FcγRIIB). FcγRIIB is the only Fc-receptor on B cells and does not bind other immunoglobulins like IgM or IgA. After juxtaposition of the BCR and FcγRIIB, the tyrosine-based inhibitory motif of FcγRIIB is in close proximity to the tyrosine-based activation motif of BCR and delivers a negative signal. **(B)** If MV-specific IgM binds to MV, it also binds via C3d to CD21 (complement receptor 2), which is part of the positively signaling CD21/CD19/CD83/Leu-13 complex. The opsonin C3d does not bind to IgG. **(C)** Interferon α (type I interferon) binds to both the interferon receptor and CD21, and the dual receptor usage leads to a strong positive signal. It stimulates antibody secretion by B cells in the presence of maternal antibodies. **(D)** A possible approach to vaccination in the presence of maternal antibodies is the reduction of the vaccine antigen into small units, which do form antigen–antibody complexes unable to cross-link BCR and FcγRIIB. An example of this approach is experimental vaccination against respiratory syncytial virus in the presence of maternal antibodies (138).

In contrast to these results, which did not support epitope masking as a mechanism, the interaction between FcγRIIB and maternal antibodies proved to be important. Both *in vitro* and *in vivo*, IgG can block B cell responses whereas F(ab)_2_ fragments cannot. A monoclonal antibody with an Fc region that does not bind to cotton rat FcγRIIB cannot inhibit B cell responses (114). These data support a mechanism of inhibition depending on complex formation of the vaccine with IgG antibodies (114). This complex cross-links the BCR (which recognizes the vaccine antigen) to the FcγIIB receptor (which binds the Fc region of the IgG antibody) on the surface of B cells. The cross-link results in a negative signal that inhibits both the proliferation of B cells (137) and the secretion of antibodies (114) (Figure 1A). In evolutionary terms, this mechanism developed to avoid an over-reactive B cell response in order to conserve resources. If IgG antibodies are already present in an organism after infection or vaccination, it is not necessary to produce more antibodies. In essence, maternal antibodies signal that there is no need to produce more antibodies. In contrast to antibody production after an active immune response, however, the passively transferred maternal antibodies decline and the infant is left without an adequate B cell and antibody response.

#### IgM stimulation of inhibited B cells

Further evidence in support of the regulatory model of B cell inhibition is provided by the role of IgM in the stimulation of B cells *in vitro* and *in vivo*. The negative feedback regulation of B cells should function in children with similar IgG titers irrespective of whether they have been actively immunized or have received maternal antibodies. However, immunized children will generate additional antibodies after re-immunization whereas children with maternal antibodies will not or at very low titers. This phenomenon can (at least partially) be explained by the presence of IgM that is being generated after active immunization. IgM forms a complex with the vaccine and a complement protein (C3d). This complex cross-links the BCR with the CR2 on the surface of B cells (Figure 1B). The cross-link results in activation of B cells *in vitro* and can partially overcome the inhibition by the cross-link of the BCR and CD32 *in vivo* (114). In consequence, some IgG antibody is produced. The important component for the stimulatory effect of CR2 is the CD21 chain that binds to C3d. This finding is consistent with the model of B cell regulation but not with epitope masking.

#### Type I interferon induction stimulates B cells in the presence of maternal antibodies

Another way to stimulate B cells experimentally in the presence of maternal antibody is the induction of type I interferon. It has been demonstrated that CD21 binds C3d and interferon alpha with the same affinity (139) indicating that interferon alpha might have a functional role in B cell stimulation. Indeed, interferon alpha stimulation leads to the up-regulation of a number of B cell genes in a human B cell line (140). *In vivo*, B cells use both the interferon receptor and CD21 (which is a chain of the CR2) as a functional interferon receptor to stimulate antibody secretion (137) (Figure 1C). One way of inducing high levels of type I interferon is the combined use of TLR-3 and TLR-9 agonists as adjuvants for immunization. Because of the dual receptor usage, the induction of type I interferon *in vivo* strongly stimulates B cell responses and restores antibody levels after immunization in the presence of maternal antibodies (137). This finding is consistent with the model of FcγRIIB-mediated inhibition of B cell regulation but not with epitope masking.

In neonates, immunization is not only impaired by the inhibitory action of maternal antibodies, but also by the overall immaturity of the immune system. The induction of type I interferon stimulates immature B cells in neonatal cotton rats, even in the presence of maternal antibodies (141).

#### Engagement of FcγRIIB is important for inhibition of B cells

Using the cotton rat model of measles virus vaccination (142), we have shown that maternal antibodies inhibit B cells through complex formation with the vaccine and cross-linking of the BCRs and FcγRIIB (114). A mixture of antibodies recognizing three or more epitopes was more inhibitory than one antibody at the same antibody concentration. These data support the current thinking that oligomerization of FcγRIIBs is required for an inhibitory signal interfering with B cell proliferation and antibody secretion (143). However, for effective oligomerization not only the number of different antibodies but also the size of an antigen seems to be important. Passively transferred antibodies specific for di-nitro-phenyl (DNP) groups inhibit immunization with DNP bound to SRBC (4–5 μm) but not immunization with DNP bound to KLH (two subunits of 30 and 33 nm) (144, 145). Similarly, measles virus, which is inhibited by maternal antibodies, is a large antigen [250–450 nm (146)] with multiple epitopes on its surface [at least 13 for hemagglutinin (MV-H) and 6 for fusion protein (MV-F)] (147). These data indicate that an antigen requires a certain size to effectively cross-link (as antibody–antigen-complex) BCRs and FcγRIIBs (Figure 1D). Consistent with this concept is a report that immunization with a small polypeptide containing a neutralizing B cell epitope escaped inhibition by maternal antibodies (138).

## Evaluation and Development of Experimental Vaccines

A number of studies claim vaccine efficacy after immunization in the presence of maternal antibodies for both approved vaccines and vaccine candidates. For experimental vaccines successes as well as failures have been reported for a variety of vector systems expressing the vaccine antigen simultaneously with or without different cytokine (combinations). The results from these studies are contradictory and it helps to clearly define the experimental conditions and study design. A vaccine can most easily be proven to be successful if it is used in the presence of low titers of maternal antibodies at the time of immunization, if T cell responses are measured and if surrogate markers like histological changes (in animal models) are used for protective efficacy. To prove convincingly vaccine efficacy in the presence of maternal antibodies, levels of maternal antibodies at the time of vaccination have to be high, neutralizing antibodies should be measured as an immunological parameter, and protection should be measured as clinical efficacy by the absence of clinical symptoms or a significant reduction in viral/bacterial titers. By these standards, very few examples of successful immunization in the presence of maternal antibodies exist, thus necessitating further research into this area. When considering the development and testing of vaccines for immunization in the presence of maternal antibodies, three options seem to emerge as being potentially successful: inoculation at a site with low IgG, continuous expression of antigen, and inoculation with adjuvants stimulating type I interferon secretion. Inhibitory maternal antibodies are of the IgG isotype, and IgG is present in respiratory and ocular fluids and saliva (148, 149) although at lower concentrations than in the blood. Occasionally, vector systems have been used successfully in animal model in the presence of maternal antibodies when alternate routes of immunization (other than subcutaneous or intramuscular) were used (134, 150–152). Experimentally, this approach could be combined with measures of IgG titers at the site of inoculation (e.g., mouth, eye, and nose) to correlate vaccination success with lower antibody titers. The concept of continuous expression of antigen is based on the finding that repeated immunization leads to an antibody response in the presence of maternal antibodies. If a vector system can be found that continuously expresses antigen, B cell responses should be stimulated when antibody titers fall to very low levels. An example of this concept is the expression of infectious bursitis virus (IBV) proteins through an attenuated Marek’s diseases virus (MDV) vector that persists in the chicken and leads to good immunity (153) in the presence of maternal antibodies. The induction of type I interferon is based on the ability of type I interferon to drive B cell responses in tissue culture and in experimental systems in the presence of maternal antibodies. An example of this concept is the immunization of pigs with adjuvants inducing type I interferon (154).

## Induction of Maternal Antibodies Through Maternal Immunization as Indirect Immunization of Infants

Because of their relative immunological immaturity, infants are usually not immunized before the age of 2–3 months (depending on country-specific immunization schedules) with the most common exception of neonatal BCG immunization in some countries. In order to provide protection earlier in life, two other vaccination strategies have been discussed and/or used: cocooning and maternal immunization. The method of cocooning relies on immunization of all people in contact with the infant to minimize the risk of pathogen transmission and infection. Although this method can be effective it is sometimes difficult to implement. Another concept that has received more attention recently is maternal immunization. Maternal antibodies have been proposed as a means of protecting the infant during a sensitive time in the development of their immune system. If protective high levels of maternal antibodies can be achieved in infants, they would be protected during the most immature phase of their immune system. In addition, the young organism becomes better adapted to cope with infectious diseases by improved non-immune host responses, e.g., faster regeneration of epithelial cells after gastro-intestinal infection. Maternal immunization has the added advantage that the mother’s immune system is fully mature and will respond well to vaccination, thus providing protection to the mother and high levels of maternal antibodies to the infant. Based on the above named studies about protective levels of maternal antibodies and their kinetics of decline, it should theoretically be possible to induce high levels of antibodies, which are protective for up to 6 months.

### Successful examples of maternal immunization

Currently, this concept is supported by data for vaccinations against tetanus, influenza virus, and pertussis. In 1961, a seminal study demonstrated the efficacy of maternal immunization with tetanus toxoid in the prevention of neonatal tetanus and reduction of neonatal mortality (155). Subsequently, the WHO Maternal–Neonatal Tetanus (MNT) elimination program has resulted in the reduction of neonatal death due to tetanus from about 787,000 death in the late 1980s to 59,000 death in 2008 (156). The final goal of the WHO campaign is to eliminate MNT, with elimination being defined at <1 case per 1000 live births.

Another case of successful maternal immunization is influenza vaccination. Influenza virus infection is a serious problem for pregnant women and immunization before, during, and after pregnancy substantially reduces serious clinical outcomes of infection. In addition, studies have demonstrated a protective effect on the child. Immunization of mothers leads to the increased transmission of maternal antibodies (157, 158), a 41–63% reduction in laboratory confirmed cases of influenza virus infection over a period of 6 months (159, 160), and a 39–91% reduction in hospitalizations of newborns (11, 159).

In addition, immunization against pertussis with the acellular pertussis antigen vaccine has proven to increase the level of maternal antibodies (161–163) and protect infants from clinical pertussis (161). A meta-analysis of vaccination studies found that cocooning (immunization of all direct contacts of an infant) and maternal immunization significantly reduced clinical disease and also were found to be cost–effective from a payer’s perspective (164). It should be noted that in these three examples inactivated vaccines were used. In principle, maternal immunization can be applied to other (live-attenuated) vaccines and infectious diseases as well. However, it has been reported that maternal immunization with a pneumococcal (polysaccharide) vaccine does not protect infants against clinical disease (165).

### Principal questions surrounding maternal immunization

In order to study the effect of maternal immunization systematically, a number of questions have to be considered. For maternal immunization to be effective, antibodies have to be the immunological correlate of protection for the specific infectious disease and a specified level of protective titers has to be known as a target parameter to have an appreciable effect on clinical outcome. The main task is to determine the best time point and immunization schedule to immunize the mother. There is little information in the literature to guide immunization of mothers in order to obtain the best transfer of maternal antibodies. Current guidelines target immunizations of women with the goal to protect them and the fetus during pregnancy, and are usually divided in immunizations before and during pregnancy. In practical terms, mothers are often being immunized only when they are pregnant and usually immunization with inactivated rather than live-attenuated vaccines is recommended. A number of studies has found that maternal antibody titers in the child are higher than in the serum of the mother when these are low (83, 157) and lower than in the serum of the mother if the mother had high levels of antibodies. The cut-off seems to be a total IgG concentration of 15 g/L (166). This phenomenon can be explained by the transport of IgG via the FcRn receptor across the placental barrier. If the FcRn receptor molecules are saturated, IgG will be degraded by lysosomal enzymes inside the vesicles (167). However, transfer of antibodies does not only depend on the total IgG concentration in the mother’s blood but also on the isotype composition. The binding affinity of FcRn is highest for IgG1, followed by IgG4, IgG3 and is weakest for IgG2 (110). In consequence, more IgG1 is transported transplacentally than e.g., IgG2. This preferential transport mechanism explains why antibodies against specific vaccines are transported more efficiently than others. Usually, antibodies against T cell-dependent antigens (proteins) are of the IgG1 isotype and are more efficiently transported than antibodies against T cell-independent antigens (polysaccharides) of the IgG2 isotype. Based on these considerations, effective vaccines could be used to immunize expecting mothers in order to protect the neonates and infants. However, it is possible that for every vaccine a threshold of maternal antibody titers can be defined that cannot be changed even with very effective vaccines. This hypothesis has to be addressed experimentally in larger clinical studies.

Another factor influencing maternal antibody titers in children is gestational age. IgG transfer from mother to child starts at 13 weeks of age. However, in the third trimester the expression of FcRn receptors increases and subsequently the transfer rate of maternal antibodies improves with the highest amount of IgG (>50%) transferred during the last 4 weeks before birth (168, 169). If relatively ineffective vaccines are available that do not induce long-lasting antibody responses, immunization during the third trimester of pregnancy might be an option. It has been demonstrated that a not very effective vaccine like pertussis might be beneficially applied during this time (170). An additional consideration for maternal immunization is the nutritional status of both mother and child. In general terms, nutrition influences immune responses but has not been shown to influence the level of antibody in the mother. However, it has been shown that undernourished children have lower maternal antibody titers, although the underlying reason is currently unknown (171). Other factors like maternal age, maternal weight, parity, and type of delivery do not influence transplacental antibody transfer (172).

In contrast to humans, immunization of agricultural animals is relatively straightforward as immunization schedules can be easily applied to breeder animals at any time. Overall, the prevailing view is that the time of immunization is not relevant as long as maternal antibody titers are high at birth because transfer occurs during a short window after birth and seems to be solely dependent on antibody titers in colostrum.

### Example: How can maternal immunization work against respiratory syncytial virus infection?

In order to answer the question of whether maternal immunization against a specific disease will lead to a reproducible and protective increase in antibody titers in children, one has to consider the total amount of IgG as well as the quality of antibody being induced by immunization. To illustrate these questions, I have chosen the example of immunization against RSV infection. RSV infection leads to severe respiratory infections in adults and is the second most common viral cause of death in the elderly (after influenza virus) (173). RSV infection is also the leading viral infection in lower respiratory disease in children, which is a particular problem in preterm babies and in neonates in their first months of life (174). So far, no vaccine or therapeutic is available. The discussion currently centers on the question of whether a vaccine could be developed that is safe and effective in neonates. Because the result of immunization of neonates is still elusive, an alternate suggestion is to use a to-be-developed vaccine to immunize mothers and provide protection for neonates and infants through maternal antibodies during the first 6 months of life. However, it is controversial as to whether maternal antibodies have a beneficial effect against RSV infection. RSV-specific antibodies are present in children during the first 6 months of life and have an estimated half-life of 1.5 months. Some studies have observed a protective effect of maternal antibodies (10, 175) whereas some did not [for review see Ref. (176)]. Currently, preterm infants (depending on their gestational age) are prophylactically treated with a monoclonal antibody against RSV. This prophylaxis is effective because the antibody has a high affinity (177, 178). In contrast to this antibody, naturally generated antibodies often have lower affinities resulting in lower neutralizing efficacy. In consequence, it will be important to induce high affinity antibodies in mothers through vaccination. Neutralizing antibodies against RSV bind to the fusion protein (F) and the glycoprotein (G). However, it has been demonstrated that vaccination with a RSV lacking the G protein is able to protect against viral challenge (179) and most attention in terms of vaccine development has been focused on the F protein. On the surface of the virion the fusion protein is folded so that the fusion peptide necessary to mediate fusion is protected from the environment (pre-fusion F). Upon triggering, the fusion protein unfolds and initiates fusion with the cellular membrane (post-fusion F). Palivizumab, the antibody that prophylactically protects children against RSV infection binds to both pre- and post-fusion F. Recent publications have defined the structure of the pre- and post-fusion F protein and have demonstrated that the most effective *in vitro* neutralizing antibodies bind to the pre-fusion F thus leading to the assumption that the goal of vaccination has to be the increase of antibody against pre-fusion F (180–182). Based on this knowledge clinical studies should be devised that not only take the total amount of antibody into account but also measure the affinity of the antibody as well as its target (G protein and pre- as well as post-fusion F protein). Antibodies are being transferred transplacentally based on their ability to bind FcRn and not on their affinity to RSV, and this transfer seems to be saturable depending on antibody level in the maternal blood. If a relatively small proportion of antibody is of high affinity it might not be possible to reach high protective levels of maternal antibodies. Another concern is immunization in the presence of maternal antibodies. As with other infectious diseases, it has been shown that even low levels of maternal antibody inhibit RSV-specific B cell and antibody responses (183, 184), a problem that will still exist after increased transfer of maternal antibodies due to maternal immunization.

## Summary and Outlook

Many examples exist that demonstrate the inhibition of both human and veterinary vaccines by maternal antibodies. B cell inhibition is mediated through a cross-link between the BCR with the FcγRIIB by a vaccine–antibody complex. Knowledge of the underlying mechanisms helps us to understand the possibilities and limitations of our current clinical approaches and will help in the development and testing of new vaccines. This knowledge also will help us to evaluate and develop the concept of maternal immunization as a mechanism to protect infants against common infectious diseases. The potential benefit of maternal immunization is protection of the mother and a delayed susceptibility to infection of the child. However, by most estimates children will not be protected for more than 6 months of life whereas full immunological maturity seems to be accomplished only after 12 months. A final unanswered question about maternal immunization, however, is how to deal with immunization in the presence of maternal antibodies. In consequence, the question of vaccinating in the presence of maternal antibodies will remain and still has to be resolved.

## Conflict of Interest Statement

The author declares that the research was conducted in the absence of any commercial or financial relationships that could be construed as a potential conflict of interest.
